# Supplementary material for: Hybrid Models and Biological Model Reduction with PyDSTool
Source: PLoS Comput Biol. 2012 Aug 9;8(8):e1002628. doi: 10.1371/journal.pcbi.1002628 (PMC3415397; doi:10.1371/journal.pcbi.1002628)
Supplement: Text S4 — Complete source code for the PyDSTool package (version 0.88.120504). Includes API documentation and help files linking to web pages. This file is identical to the current public release on Sourceforge.net. (ZIP) [file pcbi.1002628.s004.zip › PyDSTool/html/PyDSTool.Toolbox.dssrt.domscales-class.html]

xml version="1.0" encoding="ascii"?


PyDSTool.Toolbox.dssrt.domscales


| Home | Trees | Indices | Help | PyDSTool |
| --- | --- | --- | --- | --- |

Package PyDSTool ::
Package Toolbox ::
Module dssrt ::
Class domscales

[hide private]

[frames] | no frames]

# Class domscales

source code

```
object --+
         |
        domscales
```

---


|  |  |
| --- | --- |
| Instance Methods | [hide private] |

|  |  |
| --- | --- |
| \_\_init\_\_(self, focus\_var, traj\_pts, influence\_pts, influence\_type, tau\_refs, psi\_refs, opts=None)  x.\_\_init\_\_(...) initializes x; see x.\_\_class\_\_.\_\_doc\_\_ for signature | source code |

|  |  |
| --- | --- |
| calc\_epochs(self, sigma, gamma, relative\_ratios=False)  Calculate epochs without cycle checking | source code |

|  |  |
| --- | --- |
| calc\_fast\_slow(self, taus, gamma)  Determines variables which are always fast and slow, and those which are ever fast and slow, over a given set of time scale points. | source code |

|  |  |
| --- | --- |
| lookup\_index(self, ix)  Locates index ix in the epochs, returning the epoch index, epoch object, and the index within the epoch's pointset data. | source code |

|  |  |
| --- | --- |
| lookup\_time(self, t)  Locates time t in the epochs, returning the epoch index, epoch object, and the approximate corresponding index within the epoch's pointset data. | source code |

|  |  |
| --- | --- |
| calc\_regimes(self, options=None) | source code |

|  |  |
| --- | --- |
| get\_domscales\_point(self, i, relative\_ratios) | source code |

**Inherited from `object`**:
`__delattr__`,
`__getattribute__`,
`__hash__`,
`__new__`,
`__reduce__`,
`__reduce_ex__`,
`__repr__`,
`__setattr__`,
`__str__`


|  |  |
| --- | --- |
| Properties | [hide private] |

**Inherited from `object`**:
`__class__`


|  |  |
| --- | --- |
| Method Details | [hide private] |

|  |  |
| --- | --- |
| \_\_init\_\_(self, focus\_var, traj\_pts, influence\_pts, influence\_type, tau\_refs, psi\_refs, opts=None)  *(Constructor)* | source code |

x.\_\_init\_\_(...) initializes x; see x.\_\_class\_\_.\_\_doc\_\_ for
signature

Overrides: object.\_\_init\_\_: *(inherited documentation)*

|  |  |
| --- | --- |
| lookup\_time(self, t) | source code |

Locates time t in the epochs, returning the epoch index, epoch object,
and the approximate corresponding index within the epoch's pointset data.
Note, because of the time discretization, time is compared up to the next
epoch's start time.

  


| Home | Trees | Indices | Help | PyDSTool |
| --- | --- | --- | --- | --- |

|  |  |
| --- | --- |
| Generated by Epydoc 3.0.1 on Fri May 4 15:24:08 2012 | http://epydoc.sourceforge.net |
